# Oxidative-Nitrative Stress and Poly (ADP-Ribose) Polymerase Activation 3 Years after Pregnancy

**DOI:** 10.1155/2018/1743253

**Published:** 2018-08-23

**Authors:** Eszter M. Horváth, Rita Magenheim, Nóra J. Béres, Rita Benkő, Tamás Pék, Ádám G. Tabák, Csaba Szabo

**Affiliations:** ^1^Department of Physiology, Semmelweis University Faculty of Medicine, Budapest, Hungary; ^2^Institute of Human Physiology and Clinical Experimental Research, Semmelweis University Faculty of Medicine, Budapest, Hungary; ^3^Department of Medicine, Semmelweis University Faculty of Medicine, Budapest, Hungary; ^4^Department of Paediatrics, Semmelweis University Faculty of Medicine, Budapest, Hungary; ^5^Department of Pediatric Infectology, United Hospital of St. László and St. István, Budapest, Hungary; ^6^Department of Epidemiology and Public Health, University College London, London, UK; ^7^Department of Anesthesiology, University of Texas Medical Branch, Galveston, TX, USA

## Abstract

**Background:**

Oxidative-nitrative stress and poly (ADP-ribose) polymerase activation have been previously observed in healthy and gestational diabetic pregnancies, and they were also linked to the development of metabolic diseases. The aim of the present study was to examine these parameters and their correlation to known metabolic risk factors following healthy and gestational diabetic pregnancies.

**Methods:**

Fasting and 2 h postload plasma total peroxide level, protein tyrosine nitration, and poly (ADP-ribose) polymerase activation were measured in circulating leukocytes three years after delivery in women following healthy, “mild” (diet-treated) or “severe” (insulin-treated) gestational diabetic pregnancy during a standard 75 g OGTT. Nulliparous women and men served as control groups.

**Results:**

Fasting plasma total peroxide level was significantly elevated in women with previous pregnancy (B = 0.52 ± 0.13; *p* < 0.001), with further increase in women with insulin-treated gestational diabetes (B = 0.36 ± 0.17; *p* < 0.05) (*R*^2^ = 0.419). Its level was independently related to previous pregnancy (B = 0.47 ± 0.14; *p* < 0.01) and current CRP levels (B = 0.06 ± 0.02; *p* < 0.05) (*R*^2^ = 0.306).

**Conclusions:**

Elevated oxidative stress but not nitrative stress or poly (ADP-ribose) polymerase activation can be measured three years after pregnancy. The increased oxidative stress may reflect the cost of reproduction and possibly play a role in the increased metabolic risk observed in women with a history of severe gestational diabetes mellitus.

## 1. Introduction

Several previous studies examined the role of oxidative-nitrative (ON) stress and poly (ADP-ribose) polymerase-1 (PARP) activation in healthy pregnancy and in the pathogenesis of pregnancy-related complications like gestational diabetes (GDM). Blood and urinary markers of increased ON stress and PARP activation have been observed during pregnancy, and a further elevation of these parameters has been shown in gestational diabetes [1–4]. Less is known about the possible persistence of ON stress and PARP activation after delivery.

Increased formation of oxygen- and nitrogen-derived reactive species and reduced antioxidant capacities are the hallmarks of oxidative-nitrative (ON) stress. Peroxynitrite (ONOO^−^) is a reactive oxidant that is formed in the reaction of superoxide (O_2_^−^) and nitric oxide (NO) [5]. Peroxynitrite and hydroxyl radicals are potent inducers of DNA single-strand breakage, which is the obligatory trigger of the activation of the constitutive nuclear enzyme poly (ADP-ribose) polymerase-1 (PARP). PARP uses NAD^+^ as substrate to produce poly (ADP-ribose) (PAR) and binds it to several nuclear proteins (PARylation), such as histones. Overactivation results in rapid reduction of intracellular NAD^+^, leading to energy depletion, and necrotic cell death. PARP-1 also interacts with and stimulates the production of the proinflammatory nuclear factor-*κ*B (NF-*κ*B) transcription factor. Cell necrosis and proinflammatory processes further elevate ON stress, forming a vicious circle. There are various endogenous factors inhibiting the activation of PARP, for example, estrogen and the active form of vitamin D_3_ (1,25-dihydroxyvitamin D_3_) [6, 7].

The role of ON stress and consequent PARP activation in the pathogenesis of T2D and various cardiovascular disorders (atherosclerosis, hypertension, myocardial infarction, and stroke) has been well established. ON stress plays a role in the development of insulin resistance and pancreatic beta cell death and dysfunction [8–10]. The potential pathogenic role of increased ON stress and PARP activation has also been proposed during the development of various pregnancy-related complications, such as preeclampsia, GDM, or intrauterine retardation [1, 3, 4]. Increased oxidative stress can be observed even during the course of a healthy pregnancy [4].

On the other hand, the potential late consequences of pregnancy-associated ON stress and PARP activation (such as T2D and cardiovascular disease) have not yet been investigated. The aim of the present study was to examine the potential persistence of ON stress and PARP activation three years after a healthy or diabetic pregnancy in the fasting state and following a 75 g oral glucose tolerance test. In order to estimate the effect of gender and pregnancy, additional control groups of nulliparous women and men were also included. We further evaluated whether the severity of GDM (the requirement of insulin treatment) had an effect on ON stress and PARP activation. In order to assess potential covariates of ON stress and PARP activation that may link them to late metabolic complications, contemporaneous metabolic risk factors were also evaluated.

## 2. Materials and Methods

### 2.1. Participants

The participants of the present nested case-control study were recruited as a subcohort of a follow-up screening program for GDM pregnancies at the 1st Department of Medicine, Semmelweis University Faculty of Medicine (Budapest, Hungary). Forty-six women with previous GDM (diet-treated GDM (DGDM), *n* = 36; insulin-treated GDM (IGDM), *n* = 10) and 25 with normal glucose tolerance during pregnancy (PREG) agreed to participate in the sample collection for oxidative stress measurements.

To further elucidate the association between gender, previous pregnancy, and ON stress, age- and BMI-matched healthy nulliparous women (NULL, *n* = 16) and men (MALE, *n* = 15) were also investigated. All procedures of the follow-up investigation were approved by the Regional Ethics Committee of Semmelweis University (124/2007), and written informed consent was acquired from all participants.

### 2.2. Clinical Examination

The follow-up clinic consists of a clinical assessment and a 75 g oral glucose tolerance test (OGTT) with repeated blood sampling run at the 1st Department of Medicine, Semmelweis University Faculty of Medicine (Budapest, Hungary).

All participants started with an interviewer-assisted questionnaire that included sociodemographic data (age, gender, education, family income, lifestyle), general medical (medication, known illnesses, family history), and obstetrical (previous pregnancies) inquiries. For further analysis, current smoking was defined as ≥1 cigarette/day on a weekly average; educational attainment was divided into 4 categories (primary school/vocational secondary school/nonvocational secondary school/college or university degree), and family income into 5 categories (monthly income per capita <185€/185–369€/370–555€/556–740€/**>**740€). Following the questionnaire, height and weight were measured on a calibrated stadiometer in light clothing. BMI was calculated as weight (kg)/(height (m))^2^. Following fasting blood draws for glucose, insulin, HbA_1c_, C-reactive protein, 25-OH vitamin D_3_, estradiol, and ON stress determination, a standard 75 g OGTT was performed according to the WHO recommendation [11]. During the test, blood samples for glucose and insulin determinations were also collected at 30, 90, and 120 minutes after the glucose load; and for ON stress investigation at 120 minutes after glucose load.

All routine laboratory determinations were done in the Central Laboratory of Semmelweis University. Glucose levels were determined by a glucose oxidase method; serum insulin and estradiol by electrochemiluminescence immunoassay (Roche, Basel, Switzerland; Hitachi Cobas 601); HbA_1c_ by HPLC (Bio-Rad, Brussels, Belgium); C-reactive protein (CRP) by immunoturbidimetry (Beckman Coulter, Brea, CA, USA); and vitamin D_3_ by chemiluminescent immunoassay (Liaison XL, DiaSorin, Salugga, Italy).

Early insulin response (EIR: ∆insulin_30 min_/∆glucose_30min_) and area under the glucose curve (AUC glucose using the trapezoid technique) were calculated. Homeostasis model assessment insulin secretion (HOMA2B) and insulin sensitivity (HOMA2S) were determined using the HOMA calculator v2 (Diabetes Trial Units, University of Oxford, Oxford, UK).

### 2.3. ON Stress and PARP Activation

**Figure 1:**
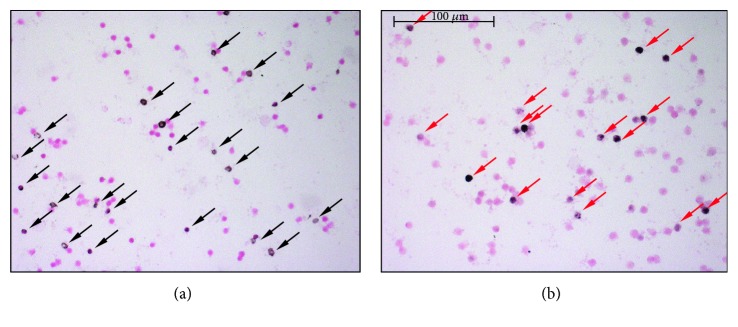
Immunostaining of leukocyte smears. (a) Representative microscopic image of leukocyte smears stained against nitrotyrosine. Black-colored precipitate indicates the diffuse labeling. Magenta-colored NFR served as counterstain. Black-colored arrows show positively stained cells. (b) Photograph of smear immunolabeled with anti-PAR antibody. Black color represents positive nuclear staining. Counterstaining was NFR. Red arrows point on positive leukocytes.

ON stress-related parameters were determined in the fasting state and 2 hours after an OGTT. Plasma total peroxide level was measured by the Oxystat kit (Biomedica, Vienna, Austria) according to the user's manual. Erroneous results due to hemolysis were excluded. To measure protein tyrosine nitration and PARP activation, circulating mononuclear leukocytes were isolated by gradient centrifugation (Histopaque-1077, Sigma/Aldrich, St. Louis, MO, USA). Protein tyrosine nitration and PARP activation were estimated by immunohistochemical labeling of methanol-fixed leukocyte smears with antinitrotyrosine (NT) (Calbiochem, Temecula, CA, USA; 1 : 80, overnight, 4°C) and anti-PAR (Calbiochem; 1 : 1000, overnight, 4°C) antibodies. Mouse and rabbit immunoglobulin-specific biotinylated antibody (Vector Laboratories, Burlingame, CA, USA) served as secondary labeling. Avidin–horseradish peroxidase complex and black-colored nickel enhanced diamino-benzidine (Vector) was used to visualize labeling; counterstaining was red-colored Nuclear Fast Red (Sigma) (Figure 1). The ratio of the positive cellular area and total cellular area was calculated by computer-based evaluation (MBFImageJ). Low cell count smears were excluded from the analysis.

### 2.4. Statistical Analysis

Descriptive statistics are given as mean ± SD for normally distributed variables and median (interquartile range) for nonnormally distributed variables. Participant characteristics by study group were compared using one-way and two-way ANOVA or Kruskal-Wallis tests with the appropriate post hoc investigations. Variables that violated the normality assumption (insulin, HOMA2B, HOMA2S, EIR, CRP, estradiol, plasma total peroxide, leukocyte NT, leukocyte PAR) were log-transformed before using them in regression models.

To investigate the association of ON stress markers and their change during OGTT with grouping variables (female sex, prior pregnancy, prior GDM, prior insulin treatment), multiple linear regression models and fixed-effect hierarchical linear specifications were used.

To explore the potential association between found predictors and ON stress parameters, the above models were further adjusted for clinical anthropological and laboratory parameters and socioeconomic data (variables presented in Table 1) if they were associated to the given ON stress parameter according to the Pearson correlation. As several gender-related parameters may alter ON stress such as estradiol and several of the variables of interest are meaningless in males such as GDM and parity, these analyses were limited to female participants.

Statistical analyses were performed by the SPSS (IBM, Armonk, NY, U.S.A.) and Graphpad Prism (Graphpad Software Inc., La Jolla, CA, USA) software. In all cases, *p* < 0.05 was considered significant.

## 3. Results

### 3.1. Characteristics of Participants at Follow-Up

The results of the anthropometric, clinical laboratory measurements, and socioeconomic data for each study group are presented in Table 1. Age, BMI, fasting glucose and insulin, and HbA_1c_ were similar in all groups, such as parity for those groups that had a prior pregnancy. Regardless of its severity, a previous diabetic pregnancy resulted in increased 120 min postload glucose levels compared to all other groups. Consequently, AUC glucose (a measure of glycemic excursion during OGTT) was significantly elevated in the previous DGDM compared to the PREG group. Differences in insulin levels were less prominent; they reached statistical significance only between the largest groups (DGDM versus PREG) in the 90-minute and 120-minute postload insulin. The prevalence of carbohydrate metabolism disorder (prediabetes and diabetes) was higher in both GDM group compared to women with prior healthy pregnancy.

No significant differences were found in measures of insulin sensitivity or secretion (HOMA-2B, HOMA-2S, and EIR) or HbA_1c_ between the groups. There were no clinically relevant differences in CRP, vitamin D_3_, or estrogen levels between any of the groups.

Twenty percent of all participants were current smokers; smoking was most prevalent among males. The majority of the participants was well educated and had an average Hungarian monthly income. Women having children had lower income per capita than nulliparous women, probably due to the higher number of dependents in the family and maternity leave. Women in the IGDM group were significantly less educated and had lower income than members of the other groups [12].

### 3.2. Predictors of ON Stress and PARP Activation in the Fasting State

**Figure 2:**
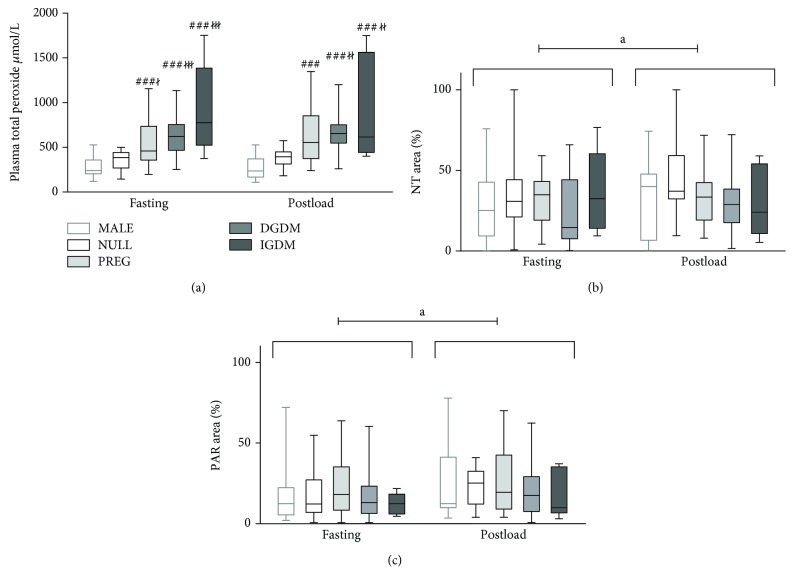
Pre- and postload values of ON stress and PARP activation markers in the study groups. (a) Plasma total peroxide. In the fasting state, all previously pregnant groups had significantly increased plasma total peroxide level compared to men and nulliparas. The two-hour postload value of this parameter was similar to that seen in the fasting state. The between-group differences in postload levels were also similar to those found in the fasting state; however, only the DGDM and IGDM groups were significantly different from nulliparas. (b) Protein tyrosine nitration in circulating mononuclear cells. There was no significant between-group difference of protein nitration neither in the fasting nor in the postload state. On the other hand, the postload values were significantly higher compared to the fasting state. (c) Protein PARylation in circulating mononuclear cells. Similar changes can be observed in the case of protein PARylation that was observed regarding protein nitration. In the boxplot, the central line shows the median; the box represents the interquartile range; and the whiskers mark the minimum and maximum values. Two-way repeated measures ANOVA, Tukey post hoc test. ###: *p* < 0.001 versus MALE; ł: *p* < 0.05 versus NULL; łł: *p* < 0.01 versus NULL; łłł: *p* < 0.001 versus NULL; a: *p* < 0.05 preload versus postload.

According to the analysis of plasma total peroxide level data with two-way ANOVA, it revealed that all previously pregnant group (PREG, DGDM, IGDM) had significantly higher fasting plasma total peroxide level compared to nulliparous women (NULL) and men (MALE) (Figure 2).

Fasting plasma total peroxide levels were further analyzed by regression models in order to identify the isolated effects of grouping variables (female sex, prior pregnancy, prior GDM, prior insulin treatment). The regression analysis suggested higher plasma total peroxide level among women (Table 2, Model 1); however, further adjustment for previous pregnancy reduced this difference to nonsignificance (Table 2, Model 2), suggesting that the observed gender difference only reflects the elevated levels among women with previous pregnancy. Prior gestational diabetes had no association with plasma total peroxide (Table 2, Model 3, 4); however, more severe cases (insulin treatment during pregnancy) presented with a significantly increased level (Table 2, Model 4, 5).

Neither tyrosine nitration nor protein PARylation was influenced by any of the above predictors (Figure 2).

### 3.3. The Effect of Contemporaneous Metabolic Risk Factors on the Association between Predictors and ON Parameters among Women

Ninety and 120 minute postload glucose levels, CRP, and monthly income were significant univariate correlates of *plasma peroxide levels*. However, according to a multivariate linear regression model—including these variables together with already identified predictors such as prior pregnancy and prior insulin treatment—both insulin treatment and monthly income became nonsignificant, and only previous pregnancy and plasma CRP levels were found to be independent determinants (*R*^2^ = 0.306, Pregnancy: B = 0.47 ± 0.14, *p* = 0.002, CRP: B = 0.06 ± 0.02, *p* = 0.015).

In a further model, we found a potential interaction between CRP and previous childbearing suggesting that increasing CRP levels is only related to higher plasma peroxide level in previously pregnant women but not in nulliparous women (*R*^2^ = 0.368, Pregnancy: B = 0.6 ± 0.15, *p* < 0.001, CRP: NS, Pregnancy ^∗^CRP: B = 0.1 ± 0.04, *p* = 0.023).

In women with previous pregnancy, vitamin D_3_ levels and EIR correlated with fasting *leukocyte nitrotyrosine levels*. According to the multiple regression model, the sole independent predictor of leukocyte nitrotyrosine levels was decreased plasma vitamin D_3_ (*R*^2^ = 0.205, B = −0.04 ± 0.01, *p* = 0.002).

Fasting *protein PARylation* was associated only with socioeconomic status (income and education). Surprisingly, having a secondary school degree was related to decreased PARP activation compared to a university degree (*R*^2^ = 0.152, B = −0.78 ± 0.36, *p* = 0.036).

### 3.4. Changes in ON Stress and PARP Activation during the OGTT

Plasma peroxide levels showed no overall change or any between-group differences during the OGTT according to our mixed model analysis.

Protein nitration increased during the OGTT overall; however, this increase was significantly smaller among those with insulin-treated GDM during pregnancy (Figure 2, Table 3).

Leukocyte *PARP activity* also increased significantly during the OGTT without any between-group differences (Figure 2, Table 3).

### 3.5. The Effect of Contemporaneous Metabolic Risk Factors on the Association between OGTT-Related Changes in ON Stress and Its Predictors

In women, the 2-hour change of plasma total peroxide level during the OGTT negatively correlated with HbA_1c_ levels while the change in protein nitration was positively related to EIR. According to the mixed (fixed effect) model, the change in plasma total peroxide level following the glucose load was negatively associated with HbA_1c_ levels (Table 4). No independent determinant was found for changes in protein nitration or PARP activation.

## 4. Conclusion

According to our present results, previous pregnancy increased the fasting level of plasma total peroxide, an oxidative stress marker 3 years after delivery, while a GDM pregnancy, per se, was not associated with elevated levels. More severe cases of GDM (those on insulin treatment during pregnancy) however showed further increases in the oxidative stress marker. Nitrative stress and PARP activation, reflected by protein nitration and PARylation in circulating mononuclear cells, were similar in all groups, suggesting that female gender, prior pregnancy, or prior GDM had no effect on these variables.

As ON stress measures were obtained only at one time point in our study, we can only speculate whether our findings reflect a persistence of the elevated oxidative stress from the time of pregnancy, or it develops later, due to other postlabor processes. In a recent study by Ziomkiewicz et al., the number of pregnancies during a lifetime was shown to have positive correlation to oxidative stress, which according to the authors' hypothesis may reflect the cost of reproductive effort in humans [13]. Similar findings were published by Mutlu and colleagues who found that total oxidant status and oxidative stress index were elevated, while total antioxidant capacity was reduced in newborns' cord blood of multiparous mothers compared to primiparous women [14]. In multiparous rats, increased oxidative stress and peroxynitrite formation was found compared to virgins, which was associated with reduced endothelium-dependent relaxation of coronary arteries and aortic rings [15]. Our results extend findings in animal models, and together with our knowledge about the role of oxidative stress in the pathogenesis of T2D, they suggest that increased oxidative stress may play a role in the association between increasing number of healthy pregnancies and the development of metabolic morbidities in women.

Several previous studies from different geographical regions examined the effect of childbearing and child-rearing on metabolic [16–19] and cardiovascular [20, 21] risk. Previous pregnancies and especially multi- and grand-multiparity were shown to have negative effects on glucose homeostasis and diabetes risk. However, lifestyle changes and socioeconomic factors were claimed to be important or even exclusive reasons for this phenomenon [16, 19]. Our results suggest that socioeconomic factors have little effect on ON stress parameters, as socioeconomic status had no effect on the pregnancy-oxidative stress association.

Given the bidirectional association between reactive oxygen species and metabolic syndrome, T2D and its complications, and our observation that more severe GDM further increases oxidative stress, we suggest the possibility of a vicious cycle from oxidative stress to the well-described elevated risk for metabolic and cardiovascular diseases of prior severe GDM. This hypothesis is further reinforced by the results of the Framingham Offspring Study, where systemic oxidative stress (urine 8-epi-prostaglandin F2*α*) was found to be in association with insulin resistance in individuals at average or elevated risk of diabetes even after adjustment for BMI [22]. Our results also correspond to the observation that insulin treatment during a GDM pregnancy (reflecting its severity) increases diabetes risk in later life [23].

Although the direct pathogenic role of CRP in T2D development has been questioned recently, it is widely accepted that it is an important marker of diabetes risk as supported by a recent meta-analysis [24]. One of the possible mechanisms leading from subclinical inflammation to the development of T2D is the elevated production of reactive oxygen and nitrogen species, which, in turn, further activates proinflammatory processes [25]. In our study, the inflammatory marker CRP was independently associated with oxidative stress, supporting this concept. The finding that increased CRP induces oxidative stress in previously pregnant women and not in their nulliparous mates may indicate the higher susceptibility of these women for subclinical inflammation.

Our finding that fasting nitrotyrosine levels correlate with early insulin response is consistent with previous studies [26, 27] showing that insulin resistance with decreasing insulin secretion leads to increased production of nitrogen-derived reactive species.

Although the exact mechanism is not fully explored, emerging evidence suggest that decreased vitamin D_3_ levels predict metabolic and cardiovascular diseases [28–31]. The association may be driven by the role of decreased vitamin D_3_ in oxidative stress, nitric oxide production, nitrative stress, and even PARP activation [32, 33]. In our participants, fasting nitrotyrosine accumulation in circulating leukocytes was negatively associated with plasma vitamin D_3_ levels and thus is in agreement with the hypothesis that vitamin D_3_ suppresses nitrative stress.

According to our data, nitrative stress and PARP activation, but not oxidative stress, were increased two hours after a glucose load. These changes were not influenced by gender, previous pregnancy, or GDM. There is an ongoing debate about the effect of acute hyperglycemia (oral glucose load) on oxidative stress and antioxidant capacity [34]. Our findings are consistent with previous studies showing that plasma nitrotyrosine levels are increased 2 hours after an OGTT, while plasma malonyl dialdehyde levels are not altered by glucose consumption [35, 36]. Although on the group level oxidative stress was not elevated due to glucose load during OGTT, HbA_1c_ levels were negatively associated with changes in oxidative stress during the OGTT. The significance and possible mechanism of this phenomenon need further investigation.

Elevated oxidative stress be measured three years after healthy pregnancy, while in case of previous insulin-treated GDM, its magnitude is even bigger. Nitrative stress and poly (ADP-ribose) polymerase activation are not altered by previous childbearing; however, they are increased by the acute elevation of plasma glucose levels. The elevation in oxidative stress that can be measured years after pregnancy may reflect the biological cost of reproduction in humans and may also play a role in the increased metabolic risk observed in women with multiple pregnancies or with a history of severe gestational diabetes mellitus.

## Figures and Tables

**Table 1:** Characteristics of participants at follow-up (^∗^: *p* ≤ 0.05 versus previous healthy pregnancy, #: *p* ≤ 0.05 versus men, ł: *p* ≤ 0.05 versus nulliparous women, ^: *p* ≤ 0.05 versus DGDM). Mean ± SEM, Median (IQR). MALE: men; NULL: nulliparous women; PREG: previous healthy pregnancy; DGDM: previous diet-treated GDM; IGDM: previous insulin-treated GDM.

	MALE	NULL	PREG	DGDM	IGDM
*N*	*n* = 15	*n* = 16	*n* = 25	*n* = 36	*n* = 10
Age (years)	35.7 ± 0.9	34.0 ± 0.5	34.1 ± 0.8	35.2 ± 0.7	36.8 ± 1.7
BMI (kg/m^2^)	27 ± 0.6	24.2 ± 1.4	23.6 ± 0.6	26.3 ± 1.0	24.6 ± 1.3
Parity		0	1 (1-2)	2 (1–3)	2 (1-2)
Level of education (1–4)	4 (3-4)	4 (3.5–4)	4 (3-4)	4 (3-4)	3 (3–3)^∗^#ł^
Income per capita (1–5)	3 (2–4)	3.5 (2.5–5)	2 (2-3)ł	2 (2-3)ł	1 (1-2)^∗^#ł^
Smoking (%)	46.7 ł^	5.9	22.2	10.5	30.8
Glucose (mmol/l)					
0 min	5.2 ± 0.2	5.3 ± 0.1	5.2 ± 0.1	5.5 ± 0.1	5.5 ± 0.2
30 min	9.3 ± 0.6	8.8 ± 0.5	7.5 ± 0.3#	9.0 ± 0.3^∗^	9.2 ± 0.6
90 min	6.5 ± 0.5	5.8 ± 0.3	5.4 ± 0.3	7.3 ± 0.3^∗^	8.3 ± 0.9^∗^ ł
120 min	4.9 ± 0.4	5.2 ± 0.3	5.3 ± 0.3	6.7 ± 0.3^∗^#ł	7.1 ± 0.8^∗^#ł
Insulin (U/ml)					
0 min	9.6 (4.7–10.9)	7.6 (3.7–10.6)	8.1 (4.5–10.1)	9.9 (7.2–14.4)	10.1 (4.6–15.3)
30 min	46.7 (35.2–96.2)	54.5 (22.8–77.7)	51.5 (30.3–67.8)	55.6 (38.9–79.0)	33.0 (22.6–86.9)
90 min	53.5 (32.9–106.8)	38.1 (21.6–55.5)	32.2 (22.9–46.0)	72.0 (38.5–97.1)^∗^	58.0 (31.5–103.0)
120 min	33.9 (6.4–65.2)	28.5 (16.0–50.0)	25.1 (14.1–38.6)	56.4 (37.1–84.8)^∗^#	45.7 (26.1–86.8)
Prediabetes (IGT + IFG) + Diabetes (%)	6.7 + 0 6.7	12.5 + 0 12.5	4 + 0 4	33.3 + 0 33.3^∗^	30 + 10 40^∗^
HOMA2B (%)	84.5 (73.5–99.6)	87.1 (67.6–97.7)	82.4 (61.8–117.2)	98.6 (80.0–130.0)	113.0 (67.4–151.9)
HOMA2S (%)	76.6 (69.5–149.8)	81.7 (69.8–189.5)	94.0 (66.4–150.0)	75.4 (51.3–104.5)	67.1 (50.9–128.1)
EIR	8.0 (5.0–15.5)	8.8 (3.5–13.1)	8.4 (4.5–10.9)	7.9 (5.8–10.4)	4.6 (1.9–14.3)
AUC glucose	1210.9 ± 122.7	1065.4 ± 42.3	1011.2 ± 37.5	1257.9 ± 40.0^∗^	1310.1 ± 111.3^∗^
HbA_1c_ (%)	5.6 ± 0.1	5.6 ± 0.2	5.4 ± 0.1	5.5 ± 0.1	5.6 ± 0.1
CRP (mg/l)	0.9 (0.3–1.9)	0.6 (0.1–1.2)	1.5 (0.3–2.2)	1.1 (0.8–3.9)ł	1.5 (0.4–4.9)
Vitamin D_3_ (ng/ml)	23.9 ± 1	23.0 ± 2.4	25.2 ± 1.8	25.9 ± 1.8	28.7 ± 3.5
Estradiol (pg/ml)	21.4 (19.0–28.2)	109.7 (66.5–224.6)#	75.4 (41.1–106.3)#	111.9 (36.9–158.2)#	82.3 (22.5–95.3)

Level of education: 1: primary school; 2: secondary school; 3: secondary grammar school; 4: college or university degree. Monthly income per capita: 1: below 185€; 2: 185–370€; 3: 370–555€; 4: 555–740€; 5: over 740€. IGT: impaired glucose tolerance; IFG: impaired fasting glucose.

**Table 2:** Potential predictors of plasma total peroxide level based on multiple linear regression with log-transformed plasma total peroxide as the outcome. To investigate the association between gender, previous pregnancy, previous GDM, GDM severity, and ON stress, a set of multiple linear regression models were built with the individual addition of dummy coded grouping variables as predictors. For the final model, only statistically significant terms were retained. Model 1: adjusted for female sex; Model 2: Model 1 + previous pregnancy; Model 3: Model 2 + previous GDM; Model 4: Model 3 + previous insulin treatment; Model 5: Model 4 with the removal of the nonsignificant GDM term. Female sex was associated with increased oxidative stress (Model 1) that was abolished by including previous pregnancy into the model (Model 2). Previous GDM did not have additional elevating effect on plasma total peroxide level upon previous healthy pregnancy (Model 3); however, previous insulin treatment itself had a positive additional impact on oxidative stress (Model 5).

Independent variable	Model 1	Model 2	Model 3	Model 4	Model 5
Female sex	0.75 ^∗∗^ (0.15)	0.29 (0.73)	0.29 (0.17)	0.29 (0.17)	0.29 (0.17)
Pregnancy		0.57 ^∗∗∗^ (0.13)	0.43 ^∗∗^ (0.15)	0.43 ^∗∗^ (0.15)	0.52 ^∗∗∗^ (0.13)
Previous GDM			0.207 (0.12)	0.15 (0.12)	
Previous insulin treatment				0.30 (0.18)	0.36 ^∗^ (0.17)
*R* ^2^	0.241	0.385	0.408	0.430	0.419

Independent variable: log plasma total peroxide, *n* = 81, values are estimated effect sizes: B (SE), (^∗^: *p* ≤ 0.05; ^∗∗^: *p* ≤ 0.01; ^∗∗∗^: *p* ≤ 0.001).

**Table 3:** Effect of OGTT and grouping variables on protein nitration and PARP activation according to mixed models with fixed effects. Difference in ON stress between the fasting and the postload state was assessed by the inclusion of the interaction between time (index) and the given predictors. As the same subjects were observed at two time points, observations are not independent (they are clustered within individuals), thus fixed-effects hierarchical linear specification was used. Both nitrative stress and PARP activity increase by the 2nd hour of the OGTT due to the glucose load (index). In case of tyrosine nitration, previous insulin treatment during index pregnancy significantly decreases the increment (index ∗ previous insulin treatment). PARP activation is not altered by the grouping variables.

Independent variable	log NT	log PAR
Dependent variable		
Index	0.33^∗∗∗^ (0.085)	0.37^∗∗^ (0.13)
Previous insulin treatment	0.08 (0.43)	
Index ∗ previous insulin treatment	−0.65^∗∗^ (0.28)	

*N* = 86 and 84, respectively. Values are estimated effect sizes: B (SE) (^∗^: *p* ≤ 0.05; ^∗∗^: *p* ≤ 0.01; ^∗∗∗^: *p* ≤ 0.001).

**Table 4:** Effect of OGTT, grouping variables, and clinical variables on plasma total peroxide level according to mixed models with fixed effects. To explore the potential associations between the identified grouping variable predictors to the changes in plasma total peroxide level in women, the mixed model was also adjusted for those variables and for their time interactions that were independent predictors of fasting plasma total peroxide level (CRP) or univariately related (Pearson correlation) to the change in plasma total peroxide level during OGTT (HbA_1c_). Including all independent determinants of plasma total peroxide level and parameters that show correlation with the change of the total peroxide level after glucose load showed that only HbA_1c_ acts as independent determinant for change in plasma total peroxide level (index ∗ HbA_1c_).

Independent variable	Estimate	Sig.
Intercept	6.11 (0.58)	0.000
Previous pregnancy	0.40 (0.13)	0.004
Previous insulin treatment	0.32 (0.17)	0.058
logCRP	0.05 (0.02)	0.015
HbA_1c_	−0.03 (0.10)	0.743
Index	0.96 (0.03)	0.002
Index ∗ HbA_1c_	−0.15 (0.05)	0.004
Index ∗ logCRP	0.01 (0.01)	0.194
Index ∗ previous pregnancy	−0.08 (0.07)	0.234
Index ∗ previous insulin treatment	−0.09 (0.08)	0.274

Dependent variable: log peroxide. *N* = 61. Values are estimated effect sizes: B (SE) (Although in the table the parameter estimate of index is positive and statistically significant, it does not mean that peroxide level actually increased between the two time points, as it refers to the counterfactual case when all interaction parameters are zero).

## Data Availability

According to ethical approval, the detailed datasets of the present study cannot be shared to a third party without permission. Permission may be asked upon individual request.
